# Association between Nonionic Amphiphilic Polymer and Ionic Surfactant in Aqueous Solutions: Effect of Polymer Hydrophobicity and Micellization †

**DOI:** 10.3390/polym12081831

**Published:** 2020-08-15

**Authors:** Samhitha Kancharla, Nathan A. Zoyhofski, Lucas Bufalini, Boris F. Chatelais, Paschalis Alexandridis

**Affiliations:** Department of Chemical and Biological Engineering, University at Buffalo, The State University of New York (SUNY), Buffalo, NY 14260-4200, USA; skanchar@buffalo.edu (S.K.); natezoyh@buffalo.edu (N.A.Z.); lucasbuf@buffalo.edu (L.B.); boris.chatelais@ensiacet.fr (B.F.C.)

**Keywords:** Poloxamer, polyethylene glycol, surfactant–polymer complex, surface tension, SANS

## Abstract

The interaction in aqueous solutions of surfactants with amphiphilic polymers can be more complex than the surfactant interactions with homopolymers. Interactions between the common ionic surfactant sodium dodecyl sulfate (SDS) and nonionic amphiphilic polymers of the poly(ethylene oxide)–poly(propylene oxide)–poly(ethylene oxide) (PEO-PPO-PEO) type have been probed utilizing a variety of experimental techniques. The polymer amphiphiles studied here are Pluronic F127 (EO_100_PO_65_EO_100_) and Pluronic P123 (EO_19_PO_69_EO_19_), having the same length PPO block but different length PEO blocks and, accordingly, very different critical micellization concentrations (CMC). With increasing surfactant concentration in aqueous solutions of fixed polymer content, SDS interacts with unassociated PEO-PPO-PEO molecules to first form SDS-rich SDS/Pluronic assemblies and then free SDS micelles. SDS interacts with micellized PEO-PPO-PEO to form Pluronic-rich SDS/Pluronic assemblies, which upon further increase in surfactant concentration, break down and transition into SDS-rich SDS/Pluronic assemblies, followed by free SDS micelle formation. The SDS-rich SDS/Pluronic assemblies exhibit polyelectrolyte characteristics. The interactions and mode of association between nonionic macromolecular amphiphiles and short-chain ionic amphiphiles are affected by the polymer hydrophobicity and its concentration in the aqueous solution. For example, SDS binds to Pluronic F127 micelles at much lower concentrations (~0.01 mM) when compared to Pluronic P123 micelles (~1 mM). The critical association concentration (CAC) values of SDS in aqueous PEO-PPO-PEO solutions are much lower than CAC in aqueous PEO homopolymer solutions.

## 1. Introduction

Multicomponent complex fluids containing surfactants and polymers are of great importance in achieving product performance in terms of interfacial properties, phase behavior, and gelation [1,2,3,4,5,6,7,8]. Surfactant–polymer formulations are used in a variety of products (e.g., foods, cosmetics, detergents, paints, and coatings) and processes (e.g., enhanced oil recovery, delivery of drugs and pesticides) [1,2]. In aqueous solutions, surfactant molecules interact with polymers above a certain concentration called the critical association (or aggregation) concentration (CAC), which is typically much lower than the critical micellization concentration (CMC) of surfactants in the absence of a polymer [1,2,9]. As the surfactant concentration increases, the polymers become saturated with bound surfactant, and free surfactant micelles begin to form above a certain polymer saturation point (PSP). The interactions between polymer and surfactant depend highly on their relative charge and hydrophobicity [2,9]. Polymers that are hydrophobically modified can self-assemble in the aqueous solution in a manner similar to low molecular weight surfactants. In the case of mixed systems comprising amphiphilic polymer and surfactant, the surfactant–polymer interactions can be rather different from those in the case of non-amphiphilic homopolymers [2,10,11,12,13]; the surfactant molecules can participate in amphiphilic polymer assemblies to form mixed micelles [2,13,14].

Water-soluble poly(ethylene oxide)–poly(propylene oxide)–poly(ethylene oxide) (PEO-PPO-PEO or EO_m_PO_n_EO_m_) block copolymers, commercially available as Pluronics (BASF Tradename) or Poloxamers or Synperonics, are used in a variety of applications including emulsions, paints and coatings, drug delivery, and nanoparticle synthesis [15,16,17,18,19,20]. PEO-PPO-PEO block copolymers are amphiphilic on the basis of their water-soluble PEO blocks and water-insoluble PPO block [21]. By varying the composition (PEO/PPO ratio) and overall molecular weight, the hydrophobic/hydrophilic properties can be varied to meet specific requirements. In aqueous solutions, PEO-PPO-PEO block copolymers form micelles with a hydrophobic core comprising PPO blocks surrounded by a corona of highly hydrated PEO blocks [22,23,24,25]. At low concentrations and temperatures in aqueous solutions, the PEO-PPO-PEO molecules are unassociated (unimers); upon an increase in the concentration or solution temperature, the PEO-PPO-PEO block copolymers associate into micelles [21,26]. The CMC and the critical micelle temperature (CMT) are related to the PEO and PPO block lengths [21]. The presence of additives such as organic solutes or solvents, electrolytes, ionic liquids, or nanoparticles greatly influences the micellization of PEO-PPO-PEO block copolymers in an aqueous solution [27,28,29,30,31,32,33,34,35].

Interesting interactions have been observed when ionic surfactants were added to aqueous PEO-PPO-PEO solutions [36,37,38,39,40,41,42,43,44,45,46,47]. The Pluronic F127 (EO_100_PO_65_EO_100_)–sodium dodecyl sulfate (SDS) system is the better studied: various types of assemblies form at different SDS/Pluronic concentrations. Pluronic P123 (EO_19_PO_69_EO_19_) has the same length PPO block but shorter PEO blocks compared to Pluronic F127, and the CMC of Pluronic P123 is much lower (by a factor of 25 in weight concentration) than that of Pluronic F127 at a given temperature [21,39,41]. Hence, it is interesting to compare the SDS–Pluronic P123 interactions to those of Pluronic F127. The mechanism of SDS association with Pluronic P123 appears similar to that of Pluronic F127; however, significant differences exist [41,42,48]. Interactions between PEO-PPO-PEO block copolymers and SDS have been investigated using calorimetry, electromotive force measurements, and light scattering [36,37,38,41,48]. Small-angle neutron scattering (SANS) studies have provided some structural information on SDS/Pluronic assemblies formed at low SDS concentrations [37,40,49]. However, the effect of polymer hydrophobicity on the structures of various SDS/Pluronic assemblies formed has not been properly resolved [37,40,41,48].

A study of the interactions between SDS and PEO-PPO-PEO block copolymers having different PEO/PPO ratios can reveal the effect of PEO and PPO groups on SDS binding to Pluronics, and provide insights on the structures of the various SDS/Pluronic assemblies formed. We report here on the interactions in the aqueous solution of SDS with Pluronic F127 (high PEO/PPO ratio) and Pluronic P123 (low PEO/PPO ratio) at block copolymer concentrations below and above the block copolymer CMC. We have employed conductivity, surface tension, pyrene fluorescence, viscosity, and SANS to understand the interactions between SDS and Pluronics. This is the first report of contrast matching SANS data at concentrations where SDS-rich SDS/Pluronic assemblies exist in solution. The organization of this paper is as follows: First, we discuss the results for SDS–Pluronic systems (i) below the CMC of the macromolecular amphiphile and (ii) above the CMC of the macromolecular amphiphile. Next, we compare the results for the two Pluronics F127 and P123.

## 2. Materials and Methods

### 2.1. Materials

Sodium dodecyl sulfate (SDS, C_12_H_25_SO_4_Na, MW = 288.4 g/mol, ≥98.5% purity) was obtained from Sigma Life Science (St. Louis, MO, USA). Deuterated sodium dodecyl sulfate (d-SDS, C_12_D_25_SO_4_Na, MW = 313.53 g/mol, 98% purity) was obtained from Cambridge Isotope Laboratories, Inc. (Tewksbury, MA, USA). Poly(ethylene oxide)–poly(propylene oxide)–poly(ethylene oxide) (PEO-PPO-PEO) triblock copolymers Pluronic F127 and Pluronic P123 were obtained from BASF Corp. (Florham Park, NJ, USA) and used as received. Pluronic F127 has a nominal molecular mass of 12600, 70% PEO, and can thus be represented as EO_100_PO_65_EO_100_ [21]. Pluronic P123 has a molecular mass of 5750, 30% PEO, and can be represented as EO_19_PO_69_EO_19_ [21].

An aqueous solution of the required PEO-PPO-PEO concentration was prepared first, and the dry surfactant was then added to this solution in order to prepare Pluronic–surfactant solutions of various surfactant concentrations. All solutions, other than those used in SANS, were prepared using milli-Q purified H_2_O (18 mΩ resistance). The samples tested by SANS were prepared in D_2_O (99.5% purity, Cambridge Isotope Laboratories, Inc., Tewksbury, MA, USA). Sufficient time was allowed for the samples to mix well and equilibrate.

### 2.2. Compositions

The CMC of PEO-PPO-PEO macromolecular amphiphiles is highly dependent on the temperature; for example, 1 °C decrease in the temperature can double the CMC [21]. Hence we considered PEO-PPO-PEO concentrations well below and above the CMC at the temperature of the experiment. The CMC values of Pluronic F127 and P123 at 25 °C are 0.7 wt% (0.555 mM) and 0.03 wt% (0.052 mM), respectively [21]. The concentrations of Pluronic F127 considered in this study are 0.01 wt% and 3 wt%, below and above the CMC, respectively, and the concentrations of Pluronic P123 below and above the CMC are 0.001 wt% and 0.5 wt%, respectively. In addition to the above polymer concentrations studied by all techniques, solutions with 0.5% Pluronic F127 were studied by conductivity and viscosity, and with 2.5% Pluronic P123 were studied by viscosity.

### 2.3. Conductivity

Addition of ionic surfactant to an aqueous solution causes the number of ions to increase and the solution conductivity to increase. Above the CMC, the increase in the solution conductivity with surfactant concentration is lower compared to that below the CMC due to the counterion association to the surfactant micelle surface. Because of this, the conductivity vs. surfactant concentration curve shows a break point at the CMC. For ionic surfactant–nonionic polymer mixed systems at fixed polymer concentration, the conductivity vs. surfactant concentration curve exhibits two break points. The first break point corresponds to the surfactant concentration where the surfactant starts binding to the polymer: critical association concentration (CAC) [50,51]. The second break point corresponds to the concentration (C_m_) where free surfactant micelles start to form in the aqueous solution, after the polymer has been completely saturated by the surfactant [50,51].

An Accumet XL 50 conductivity meter with potassium chloride electrode was used to measure the conductivity of aqueous SDS–Pluronic F127 or P123 systems. In the conductivity vs. surfactant concentration plots for SDS–Pluronic F127 or P123 systems, two linear regions with one break point were observed, instead of the two break points outlined above [50]. The lower concentration break point has not been observed here because SDS binds to Pluronic F127 or Pluronic P123 at SDS concentrations (<<1 mM) that are very low relative to the SDS concentration range (1–20 mM) considered in our conductivity plots.

### 2.4. Surface Tension

When surfactant is added to an aqueous solution the surface tension decreases, and above the CMC, it reaches an almost constant value or changes with a low slope. In surfactant–polymer systems at a fixed polymer concentration and varying surfactant concentration, the changes in surface tension reflect the surfactant and polymer interactions [37].

The surface tension of aqueous SDS solutions in the presence of Pluronic F127 or Pluronic P123 has been measured using a Kruss K100 force tensiometer by employing the Wilhelmy plate method [52]. The volume of sample used for the measurement was ~20 mL, and the Wilhelmy plate was left in contact with the sample for about 600–900 s.

### 2.5. Micropolarity

Pyrene is hydrophobic, and given the opportunity, it tends to move from the aqueous phase to a hydrophobic environment. The monomer emission spectrum of pyrene exhibits a vibronic fine structure, with the ratio of the intensities of the first and third vibronic peaks (I1/I3) depending strongly on the polarity of the microenvironment experienced by pyrene [53,54]. Information on the critical concentrations for different Pluronic–surfactant solutions was obtained here from the point where I1/I3 starts to decrease following a plateau region in the I1/I3 vs. surfactant concentration curve. Small differences between critical concentrations values obtained by different techniques may be due to small variation in the temperatures at which various experiments were performed. The micellization of PEO-PPO-PEO block copolymers is not abrupt but takes place over a concentration and temperature range [21,26].

Pyrene (Fluka, Buchs, Switzerland) dissolved in ACS/USP grade ethanol was used as a probe of micropolarity [54], and 2 μL of 1 mM pyrene (Fluka, Buchs, Switzerland) in ethanol (ACS/USP grade) was added to 3 g sample solutions. The resulting overall pyrene and ethanol concentrations were about 0.7 μM and 6.7 × 10^−4^ vol %, respectively. A Hitachi F-2500 fluorescence spectrophotometer was used for pyrene fluorescence studies of aqueous SDS solutions in the absence and in the presence of Pluronic F127 or P123. The excitation wavelength of pyrene was λ = 335 nm. The intensity was recorded in the 340–460 nm emission wavelength range.

### 2.6. Viscosity

Viscosity measurements of aqueous polymer–surfactant solutions can discern possible changes in the polymer conformation [55,56]. Viscosity measurements of SDS in aqueous solutions in the absence and in the presence of Pluronic F127 or P123 were performed using a Cannon-Fenske capillary viscometer (size 50). The kinematic viscosity (η) is calculated by multiplying the efflux time with the viscometer calibration constant provided by the manufacturer, Cannon Instrument Co. (State College, PA, USA). The relative viscosity (η_r_ = η/η_0_) of a solution is calculated from the ratio of the kinematic viscosity of the solution (η) and the kinematic viscosity of the solvent (η_0_).

For the aqueous SDS + Pluronic solutions studied as a function of SDS concentration at a fixed Pluronic concentration, the relative viscosity is calculated considering the solvent to be either plain water or aqueous Pluronic solution. The relative viscosity values at a particular surfactant concentration can be compared better between different SDS + Pluronic systems when the solvent is considered to be water. The relative viscosity trend as a function of surfactant concentration is better understood when the solvent is considered to be the Pluronic aqueous solution.

For the SDS + Pluronic F127 system, viscosity data have been collected at 20 °C (where CMC = 1–2 wt% [57]) for 0.01 wt% Pluronic F127 (well below the CMC), 0.5 wt% (below CMC) and 3 wt% (above CMC, as also attested by the observed very different viscosity trend compared to that below CMC concentrations). For SDS + Pluronic P123, viscosity data have been collected at 18.5 °C (where CMC = 0.3 wt% [21]) for 0.001 wt% Pluronic P123 (well below the CMC), 0.5 wt% (above the CMC but close to CMC), and 2.5 wt% (well above the CMC). The analysis/interpretation of results is not affected by the small differences in temperature between viscosity and other techniques.

### 2.7. Small-Angle Neutron Scattering (SANS)

SANS utilizes scattering of neutrons at small scattering angles to probe the material structure at the nanometer to micrometer scale. SANS is an appropriate technique to characterize aqueous polymer or surfactant solutions and has been widely used to determine the size and structure of PEO-PPO-PEO amphiphilic block copolymer micelles or low-molecular-weight surfactant micelles [22,29,58,59]. The large difference in the scattering lengths of hydrogen and deuterium provides a good contrast to reveal the structures formed by hydrogenous molecules in D_2_O solvent.

SANS measurements of aqueous polymer and surfactant solutions were performed on the NG-7 and NG-B 30 m SANS instruments at the Center for Neutron Research (NCNR), National Institute of Standards and Technology (NIST), Gaithersburg, MD. Neutrons with 6 Å wavelength and wavelength spread (Δλ/λ) of 12% were focused on samples kept in quartz cells of 1 mm, 2 mm, or 4 mm thickness. Sample-to-detector distances (SDD) of 2, 6.5 and 13 m, or 1.33, 4 and 13.17 m were used for each sample in order to span the wave vector (q) range 0.05 Å^−1^ < q < 0.5 Å^−1^. The measurement time was in the range 180–4200 s. Reduced SANS data of a particular sample at three SDD were combined into one data file, after trimming data points from the ends of each set and rescaling the overlap regions [60]. In the data reduction process, scattering intensity raw data were corrected for the scattering from empty cell, background, and detector sensitivity and converted to an absolute intensity scale [60]. The scattering contribution from the solvent has been accounted for by fitting a straight line to the solvent intensity data in the high q range (to avoid the influence of noisy data) and subtracting the intensity of this straight line from the sample scattering intensity. The fraction of the solvent scattering intensity subtracted is the volume fraction of solvent in the sample.

## 3. Results and Discussion

In what follows, we present results from aqueous SDS + PEO-PPO-PEO systems, first below and then above the CMC of the macromolecular amphiphile, followed by a comparison of the systems containing the two macromolecular amphiphile at conditions below and above the Pluronic CMC. In each subsection, we present the interactions of SDS with Pluronic F127 first, followed by the interactions of SDS with Pluronic P123. This is because the SDS + Pluronic F127 mode of association changes over a relatively wide SDS concentration range, making it clearer to interpret these experimental results, compared to the SDS + Pluronic P123 system.

### 3.1. Below the CMC of Macromolecular Amphiphile

#### 3.1.1. Pluronic F127 Systems

**Figure 1:**
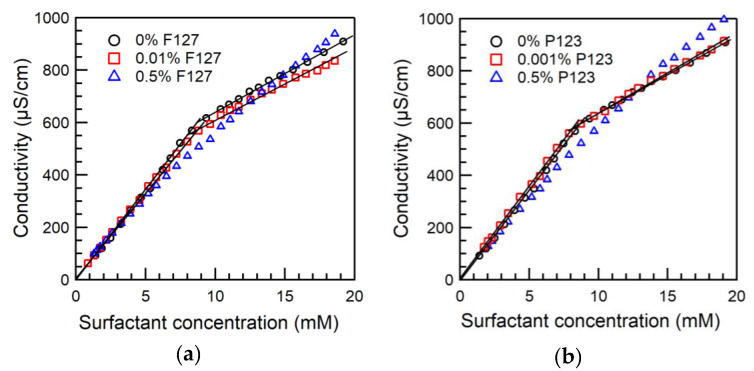
Conductivity of SDS aqueous solutions in the absence and in the presence of (**a**) 0.01% or 0.5% Pluronic F127 and (**b**) 0.001% or 0.5% Pluronic P123, plotted as a function of surfactant concentration (22 °C).

Conductivity of the aqueous SDS + Pluronic F127 solutions is plotted in Figure 1a as a function of SDS concentration. In the absence of PEO-PPO-PEO polymers, the CMC for SDS in the aqueous solution determined from conductivity is 8.7 mM. In the SDS + 0.01% Pluronic F127 system, a change in the slope is observed at 8.5 mM SDS, a concentration that likely corresponds to the surfactant concentration (C_m_) where free SDS micelles form in the aqueous solution.

**Figure 2:**
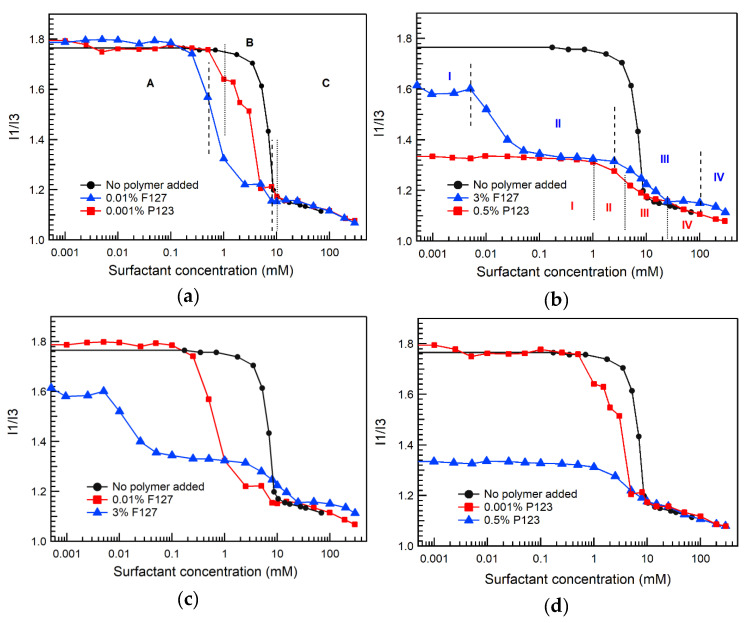
Pyrene fluorescence intensity I1/I3 ratio of SDS aqueous solutions in the absence and in the presence of 0.01% Pluronic F127, 3% Pluronic F127, 0.001% Pluronic P123, or 0.5% Pluronic P123, plotted as a function of SDS concentration (22 °C); (**a**) both Pluronics below their CMC; (**b**) both Pluronics above their CMC; (**c**) Pluronic F127 systems; (**d**) Pluronic P123 systems. The vertical dotted lines indicate SDS concentration regions that correspond to different stages of PEO-PPO-PEO block copolymer and ionic surfactant interactions, as described in the Results and Discussion Section and depicted in Figure 6.

Pyrene fluorescence spectroscopy results for SDS in water and in 0.01% Pluronic F127 aqueous solutions at 22 °C are shown in Figure 2a,c. In the absence of added block copolymer, the I1/I3 ratio dropped steeply as a function of surfactant concentration with an inflection point at around 8 mM SDS, matching well the SDS CMC value obtained by other techniques [59]. At SDS concentrations below 0.1 mM, the I1/I3 ratio in the SDS + 0.01% Pluronic F127 system remained constant at a value that is almost equal to that of I1/I3 in water, indicating that pyrene is located in an aqueous environment and no hydrophobic domains form below 0.1 mM SDS. The I1/I3 ratio started to decrease above 0.1 mM SDS, indicating that SDS starts to bind with unassociated Pluronic F127 molecules to form SDS-rich SDS/Pluronic F127 assemblies that offer hydrophobic domains for pyrene to partition in. The I1/I3 ratio reached a constant value at 2.5–5 mM SDS, likely due to the saturation of Pluronic F127 with SDS. Above 5 mM SDS, the I1/I3 ratio deceased again and reached a plateau at 8 mM. Above 8 mM, the I1/I3 ratio matches that of SDS micelles in plain water, suggesting the formation of free SDS micelles (C_m_).

For the SDS + 0.01% Pluronic F127 system considered here, the SDS/Pluronic F127 molecular ratio at saturation is 250 (estimated from the difference between the concentration where SDS starts binding to Pluronic F127 and the concentration where Pluronic F127 becomes saturated with SDS). From the CAC and PSP values reported in the literature [36], we calculated the number of SDS molecules per Pluronic molecule at saturation, which varied from 5 (0.5% F127 at 35 °C) to 1200 (0.1% F127 at 35 °C). Such binding ratios for SDS–Pluronic systems have not been previously reported, other than by Hecht & Hoffmann [61,62]. Low Pluronic concentrations give much higher SDS/Pluronic molecular ratios; such high numbers are not consistent with saturation at the single polymer chain level but rather at a level of the overall solution.

**Figure 3:**
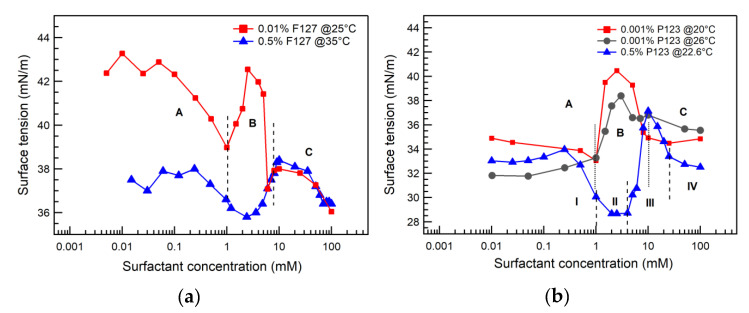
Surface tension of SDS aqueous solutions in the presence of (**a**) 0.01% Pluronic F127 (at 25 ± 0.6 °C), 0.5% Pluronic F127 (at 35 °C, micellized), or (**b**) 0.001% Pluronic P123 (at 20 °C and at 26 ± 0.6 °C), 0.5% Pluronic P123 (at 22.6 °C), plotted as a function of the SDS concentration. The SDS + 0.5% Pluronic F127 surface tension data are from the literature [37]. The vertical dotted lines indicate SDS concentration regions that correspond to different stages of PEO-PPO-PEO block copolymer and ionic surfactant interactions, as described in the Results and Discussion section and depicted in Figure 6.

The surface tension of SDS + 0.01% Pluronic F127 solutions at 25 °C is shown in Figure 3a,c. The surface tension of 0.01% Pluronic F127 aqueous solution in the absence of surfactant is 40 mN/m. Below 1 mM SDS, the surface tension of the SDS + 0.01% Pluronic F127 solution decreased upon increasing SDS concentration due to the adsorption of SDS molecules at the air/water interface [37]. In the 1–2.5 mM SDS concentration range the surface tension increased; this can be ascribed to the binding of SDS to unassociated Pluronic F127 molecules to form SDS-rich SDS/Pluronic F127 assemblies that have a polyelectrolyte [59] nature and tend to desorb from the air/water interface into the bulk solution, thus increasing the surface tension [37]. In the 2.5–8 mM SDS concentration range, the surface tension decreased, due to saturation of Pluronic F127 with SDS and increasing concentration in unassociated SDS molecules, which adsorb at the air/water interface and decrease the surface tension. Above 8 mM SDS, the SDS + 0.01% Pluronic F127 surface tension decreases gradually, consistent with the formation of free SDS micelles in the aqueous solution above the C_m_ concentration [37]. Similar surface tension behavior of SDS solutions in the presence of unassociated Pluronic F127 has been reported previously [37].

**Figure 4:**
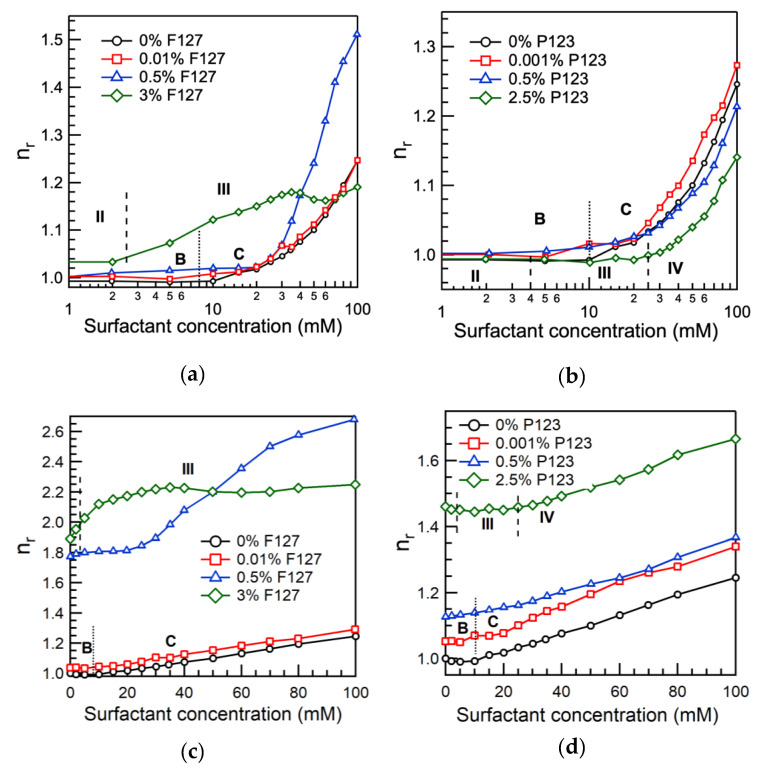
Relative viscosity as a function of surfactant concentration in the aqueous solution in the absence of added polymer and in the presence of (**a**,**c**) 0.01%, 0.5%, or 3% Pluronic F127 (20 °C) or (**b**,**d**) 0.001%, 0.5%, or 2.5% Pluronic P123 (18.5 °C). In Figure 4a,b, the relative viscosities (η_r_ = η/η_0_) are calculated considering η_0_ as the viscosity of the corresponding aqueous Pluronic solution. In Figure 4c,d, relative viscosities are calculated considering η_0_ as the viscosity of plain water. The vertical dotted lines indicate SDS concentration regions that correspond to different stages of the PEO-PPO-PEO block copolymer and ionic surfactant interactions, as described in the Results and Discussion Section and depicted in Figure 6.

We checked the solution viscosity for indications of possible changes in the SDS/Pluronic F127 assemblies. The relative viscosity of SDS in water and in 0.01% or 0.5% Pluronic F127 aqueous solutions is plotted in Figure 4a,c as a function of surfactant concentration. The relative viscosity increased monotonically with SDS concentration for all three systems. The relative viscosity of SDS + 0.01% Pluronic F127 solution is similar to that of SDS in plain water; however, the relative viscosity of SDS + 0.5% Pluronic F127 solution above 25 mM SDS is much higher when compared to the other two systems, consistent to an extended conformation in the SDS-rich SDS/Pluronic F127 assemblies due to their polyelectrolyte nature. The greater number of PEO-PPO-PEO molecules that are present in the SDS + 0.5% Pluronic F127 system has been accounted for in the way that the relative viscosity has been calculated in Figure 4a.

In order to organize the results from different techniques (Figure 2 and Figure 3), we demarcated SDS concentration regions (A, B, and C) of different surfactant+polymer interaction mode. In region A (<0.5 mM SDS), SDS molecules and Pluronic F127 unimers compete to adsorb at the air/liquid interface. In region B (0.5–8 mM), SDS forms SDS-rich SDS/Pluronic assemblies with unassociated (unimer) Pluronic F127 until the polymer reaches saturation with SDS. In region C (>8 mM), free SDS micelles form in aqueous solution. These surfactant concentrations are in good agreement with reported electromotive force (EMF) and isothermal titration calorimetry (ITC) results [36,41]. Surface tension data of SDS + 0.5% Pluronic F127 at 15 °C have suggested that the CAC of SDS and 0.5% unassociated Pluronic F127 was at 0.35 mM SDS, and the PSP was at 35 mM [37]. This CAC value is in agreement with our results, but the PSP is very high when compared to our result of 2.5 mM. The reported [37] surface tension trend with SDS concentration is similar to our surface tension data. We considered here the PSP to be the SDS concentration where the surface tension starts to decrease after the SDS/Pluronic F127 complex formation. In a previous report [37], the PSP of SDS + 0.5% unassociated Pluronic F127 at 15 °C was considered as the intersection point of surface tension curves of two systems, SDS + 0.5% unassociated Pluronic F127 at 15 °C and SDS + 0.5% micellized Pluronic F127 at 35 °C.

#### 3.1.2. Pluronic P123 Systems

Conductivity data for SDS + 0.001% Pluronic P123 solutions are presented in Figure 1b. A change in the slope of conductivity vs. surfactant concentration curve is observed at 8.5 mM (Figure 1b), which corresponds to the concentration where free SDS micelles form (C_m_).

Pyrene fluorescence intensity I1/I3 ratio values for SDS + 0.001% Pluronic P123 aqueous solutions are presented in Figure 2a,d. The I1/I3 ratio started to decrease above 0.5 mM SDS, indicating that SDS starts to bind with unassociated Pluronic P123 molecules to form SDS-rich SDS/Pluronic P123 assemblies. The I1/I3 ratio reached a constant value at 10 mM SDS; above this concentration the I1/I3 ratio matches that of SDS in plain water, suggesting the formation of free surfactant micelles in the 0.001% Pluronic P123 solution.

The surface tension of SDS + 0.001% Pluronic P123 aqueous solutions at 20 °C is shown in Figure 3b,d. The surface tension of aqueous 0.001% Pluronic P123 in the absence of surfactant is 35 mN/m. After remaining at this level, the SDS + 0.001% Pluronic P123 surface tension increased above 1 mM SDS, reached a maximum at 2.5 mM SDS, and then decreased upon further increase in SDS concentration. The higher surface tension in the 1–2.5 mM SDS range can be ascribed to the formation of SDS-rich SDS/Pluronic P123 assemblies with a polyelectrolyte nature [37,59]. The decrease in surface tension above 2.5 mM SDS can be ascribed to Pluronic P123 becoming saturated with bound SDS and increasing SDS concentration at the air/water interface. Above 10 mM SDS, the SDS + 0.001% Pluronic P123 surface tension remained constant, suggesting the formation of free SDS micelles in the aqueous solution, in agreement with the C_m_ value determined from our conductivity and pyrene experiments.

The relative viscosity of the SDS + 0.001% Pluronic P123 aqueous solutions increased monotonically with an increase in the surfactant concentration, and is close to that of aqueous SDS solutions in the absence of added polymer (Figure 4b,d).

Similar to the SDS + 0.01% Pluronic F127 system, in the SDS + 0.001% Pluronic P123 system three SDS concentration regions can be identified, A (below 1 mM SDS), B (1–10 mM), and C (above 10 mM), where the mode of association is the same as described previously for the SDS + 0.01% Pluronic F127 system. In agreement with our results, ITC experiments have shown that, at 15 °C, SDS binds to 0.1% unassociated Pluronic P123 at 0.3 mM SDS [41].

### 3.2. Above the CMC of Macromolecular Amphiphile

#### 3.2.1. Pluronic F127 Systems

Pyrene fluorescence intensity I1/I3 ratios of SDS + 3% Pluronic F127 aqueous solutions are presented in Figure 2b,c. In the absence of added SDS, the I1/I3 ratio of 3% Pluronic F127 aqueous solution is 1.56—lower than the I1/I3 ratio of pyrene in plain water (1.8)—because pyrene is localized in the Pluronic F127 micelles. The I1/I3 ratios of SDS + 3% Pluronic F127 solutions started to decrease at 0.005 mM SDS, which is ascribed to the binding of SDS to Pluronic F127 micelles to form Pluronic-rich SDS/Pluronic F127 assemblies [36,37]. The I1/I3 ratio attained a constant value at 0.05 mM SDS and remained constant until 2.5 mM SDS. At 2.5 mM SDS, the I1/I3 ratio started decreasing again until it reached a plateau at 25 mM SDS. The decrease in I1/I3 ratio at 2.5 mM SDS is attributed to the binding of SDS molecules to unassociated Pluronic F127 molecules to form SDS-rich SDS/Pluronic F127 assemblies [36,37]. The I1/I3 ratio remained constant at 25–100 mM SDS, and then decreased slightly again. The I1/I3 ratio of SDS + 3% Pluronic F127 system above 100 mM SDS is the same as the I1/I3 ratio of micellar SDS solution in water in the absence of additives. This indicates that the decrease in I1/I3 ratio at 100 mM SDS is due to the formation of free SDS micelles in the aqueous solution.

For SDS–Pluronic aqueous solutions above the CMC of the amphiphilic polymer, the SDS present in the aqueous solution is mostly associated with the polymer in the SDS concentration range where conductivity was measured. Consequently, the change in conductivity vs. surfactant concentration for the SDS + 0.5% Pluronic F127 system is rather gradual (Figure 1a), and a precise SDS concentration where the slope changes could not be identified. Figure 1a indicates that the conductivity of SDS + 0.5% Pluronic F127 solutions at high SDS concentrations is greater than that of SDS in plain water (no polymer), suggesting higher counterion dissociation in SDS-rich SDS/Pluronic assemblies. This constitutes a difference between PEO-PPO-PEO and PEO homopolymer systems, where the fractional charge of an SDS molecule in a PEO-bound micelle was found ~40% lower compared to the case of polymer-free SDS micelles [59].

The relative viscosity (η_r_ = η/η_0_) of SDS + 3% Pluronic F127 aqueous solutions is calculated considering as solvent viscosity (η_0_) either the viscosity of the corresponding aqueous Pluronic solution (3% F127 + water) (Figure 4a) or the viscosity of pure water (Figure 4c). In both η_0_ cases, upon SDS addition to Pluronic F127 micellar solution (3% F127), the viscosity increases steeply due to a breakdown of the nonionic block copolymer micelles after being charged with the ionic surfactant. The relative viscosity of SDS + 3% Pluronic F127 aqueous solutions increased until a certain SDS concentration, and above, it did not change much. At 35–100 mM SDS, the relative viscosity remained almost the same; this surfactant concentration range corresponds to region IV (see below), where SDS-rich SDS/Pluronic F127 assemblies are present as ascertained from our pyrene fluorescence results.

The volume fraction ϕ_hydr_ of hydrated SDS + Pluronic assemblies can be obtained by fitting the general equation for the viscosity of a dispersion of “particles” having ϕ volume fraction, η_r_ = 1 + 2.5ϕ, to the relative viscosity vs. surfactant concentration data calculated using as the solvent viscosity (η_0_) and the corresponding aqueous Pluronic solution viscosity. The volume fraction of the water hydrating the SDS + Pluronic assemblies is obtained from the difference [ϕ_hydr_ - ϕ_dry_], where ϕ_dry_ reflects the volume of the “dry” (not hydrated) surfactant and Pluronic present in the solution. Dry volume fractions of SDS and Pluronic have been calculated from the SDS and Pluronic molar concentrations (number of molecules) and the molecular volume of SDS (439.5 Å^3^) and Pluronic (19198.8 Å^3^ for F127 and 9081.6 Å^3^ for P123). The volume of SDS and Pluronic molecules have been obtained by adding the volumes of individual molecular units (CH_3_, CH_2_, SO_4_, and Na for SDS, and C_2_H_4_O and C_3_H_6_O for Pluronic F127 and P123) [59,63]. Changes in the polymer conformation were assessed through changes (increases or decreases) in the hydration of SDS/Pluronic assemblies. For ~10% increase in relative viscosity (upon a ~350% change in ϕ_dry_), both ϕ_hydr_ and [ϕ_hydr_ - ϕ_dry_] increased by ~350% for SDS + 0% Pluronic F127 solutions. For ~10% increase in relative viscosity (~210% change in ϕ_dry_), both ϕ_hydr_ and [ϕ_hydr_ - ϕ_dry_] increased by ~210% for SDS + 0.01% Pluronic F127 solutions. In the case of SDS + 0.5% Pluronic F127 solutions, for a ~10% increase in relative viscosity (due to ~35% change in ϕ_dry_), ϕ_hydr_ increased by ~60% and [ϕ_hydr_ - ϕ_dry_] increased by ~64%. The relative increase in ϕ_dry_, ϕ_hydr_, and [ϕ_hydr_ - ϕ_dry_] is the same for 0% or 0.01% Pluronic F127, indicating that hydration does not change. Whereas in the case of 0.5% Pluronic F127, for a 35% increase in ϕ_dry_, the increase in ϕ_hydr_ is 60% and in [ϕ_hydr_ - ϕ_dry_] is 64%. This greater increase (doubling) in ϕ_hydr_ or [ϕ_hydr_ - ϕ_dry_] with respect to ϕ_dry_ in this system intimates a more extended conformation when Pluronic molecules participate in SDS-rich SDS/Pluronic assemblies. Pluronic molecules also form SDS-rich SDS/Pluronic assemblies in the SDS + 0.01% Pluronic F127 system; however, since the Pluronic concentration in this system is low, any relative increase in [ϕ_hydr_ - ϕ_dry_] is too small to discern.

On the basis of the above results for the SDS + 3% (fixed) Pluronic F127 system, four SDS concentration regions are demarcated. In region I (< 0.005 mM SDS), there is no detectable association between SDS molecules and Pluronic F127 micelles. In region II (0.005–2.5 mM), SDS associates with Pluronic F127 micelles to form Pluronic-rich SDS/Pluronic F127 assemblies; these decrease in size and Pluronic F127 association number with increasing SDS concentration. In region III (2.5–100 mM), SDS associates with Pluronic F127 unimers to form SDS-rich SDS/Pluronic F127 assemblies. In region IV (>100 mM), free SDS micelles form in the aqueous solution.

In agreement with the results reported here, EMF and light scattering for SDS + 3% Pluronic F127 at 25 °C have shown the binding of SDS to Pluronic F127 micelles to occur even at the lowest measured SDS concentration (0.01 mM), and SDS-rich SDS/Pluronic F127 assemblies to start forming at 2 mM SDS [36]. Surface tension data for SDS + 0.5% Pluronic F127 at 35 °C (micellized F127) have shown the breakdown of Pluronic-rich SDS/Pluronic F127 assemblies to commence at ~0.25 mM SDS, and the SDS-rich SDS/Pluronic F127 assemblies to start forming at around 2.5 mM, in agreement with our results [37]. At 0.05–2.5 mM SDS, where breakdown of the Pluronic-rich SDS/Pluronic F127 assemblies takes place [36], we found the polarity reported by pyrene not to change. Light scattering and SANS results have shown that, for the SDS + 3% Pluronic F127 system in the 2–15 mM SDS range, breakdown of Pluronic-rich SDS/Pluronic assemblies takes place together with the formation of SDS-rich SDS/Pluronic assemblies [36,62]. ITC results [36,41] have shown an exothermic peak when SDS binds to Pluronic micelles, indicative of the breakdown of Pluronic micelles (Pluronic micelle formation is endothermic) [21,28,31]. Light scattering intensity, which according to the authors “monitors the size of only the F127 component of the [SDS/Pluronic assemblies]”, decreased and reached a plateau at higher (>10 mM) SDS concentrations, consistent with a breakdown (decrease in the size) of Pluronic micelles [36]. SANS data for SDS + 3% Pluronic F127 show a transition in the scattering pattern with increasing SDS concentration [62]. At low SDS concentrations, the SDS + 3% Pluronic F127 scattering pattern is similar to that of 3% Pluronic F127 in water (no surfactant present), whereas, at high SDS concentrations, the scattering pattern is similar to that of SDS micelles in water (no polymer present) [62]. All these results confirm that, with increasing SDS concentration, Pluronic-rich SDS/Pluronic F127 assemblies break down and form SDS-rich SDS/Pluronic F127 assemblies.

The I1/I3 ratio of SDS + 3% Pluronic F127 solutions decreased when SDS started binding to Pluronic F127 micelles (region II) and when SDS-rich SDS/Pluronic F127 assemblies formed (region III), indicating that the pyrene microenvironment is becoming more hydrophobic with SDS addition. This also suggests that the SDS-rich SDS/Pluronic F127 assemblies are more hydrophobic than Pluronic-rich SDS/Pluronic F127 assemblies in region II. The structure of Pluronic-rich and SDS-rich SDS/Pluronic F127 assemblies has not been resolved in the literature. A SANS study on SDS + 3% Pluronic F127 solution has reported the Pluronic F127 association number and size for Pluronic-rich SDS/Pluronic F127 assemblies, but not the SDS association number or location (i.e., inside the mixed micelle core or in the corona) in Pluronic-rich SDS/Pluronic F127 assemblies [37]. This article did not report the SANS intensity data and the corresponding fits for Pluronic-rich SDS/Pluronic F127 assemblies, hence there is no opportunity for independent confirmation of the quality of the fits and the proposed structure [37]. This SANS study also reported fitted parameters for 100 mM SDS (hydrogenous) + 3% Pluronic F127 (at 36.5 °C) assuming a core-shell structure [37]. However, contrast matching with deuterated SDS was not done at this concentration where SDS-rich SDS/Pluronic assemblies exist in the solution, and important parameters such as hydration were not reported [37].

**Figure 5:**
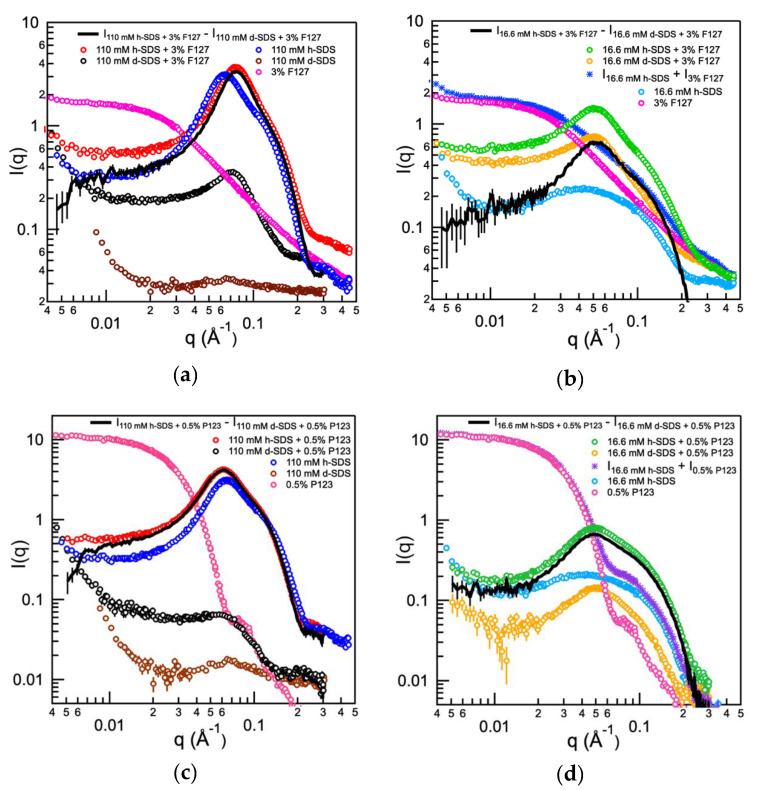
SANS absolute intensity profiles of SDS in D_2_O, in 3% Pluronic F127 in D_2_O, and in 0.5% Pluronic P123 in D_2_O solutions at 22 °C, corrected for D_2_O scattering. The intensity of 16.6 mM h-SDS is added to the intensity of 3% Pluronic F127 (**b**) or 0.5% Pluronic P123 (**d**) (all three have been corrected for D_2_O scattering) and compared with the measured intensities for mixtures of 16.6 mM h-SDS in 3% Pluronic F127 or 0.5% Pluronic P123. The intensity of (**a**) 110 mM or (**b**) 16.6 mM d-SDS + 3% Pluronic F127 is subtracted from the intensity of 110 mM or 16.6 mM h-SDS + 3% Pluronic F127, respectively, and the intensity of (**c**) 110 mM or (**d**) 16.6 mM d-SDS + 0.5% Pluronic P123 is subtracted from the intensity of 110 mM or 16.6 mM h-SDS + 0.5% Pluronic P123, respectively, and the resulting intensity is compared with the intensity from 110 mM or 16.6 mM h-SDS (no polymer present).

We employed SANS with contrast matching in order to obtain direct evidence of Pluronic-rich and SDS-rich SDS/Pluronic F127 assembly structure in the aqueous solution. SANS intensity has been recorded for aqueous SDS solutions (no polymer added), aqueous Pluronic solutions (no surfactant present), and SDS–Pluronic mixtures in D_2_O with either hydrogenous or deuterated SDS, both at the same molar composition. Deuterated SDS (d-SDS) is used in order to contrast match the solvent D_2_O scattering length density and obtain the structural information of the hydrogenous PEO-PPO-PEO block copolymers participating in SDS/Pluronic assemblies. When hydrogenous SDS (h-SDS) is used, structural information of the entire SDS/Pluronic assemblies is obtained. According to Figure 5a,b, the scattering from 3% Pluronic F127 does not show a correlation peak in the absence of ionic surfactant; however, a strong correlation peak emerges upon addition of 16.6 mM or 110 mM hydrogenous SDS (h-SDS). The 16.6 mM SDS + 3% Pluronic F127 composition falls in region III with SDS-rich SDS/Pluronic F127 assemblies. The 110 mM SDS + 3% Pluronic F127 composition falls in region IV where SDS-rich SDS/Pluronic F127 assemblies exist along with free SDS micelles.

The overall scattering intensity from 110 mM or 16.6 mM h-SDS + 3% Pluronic F127 includes contributions from both surfactants and polymers in solution. We have summed the scattering profiles originating from 110 mM or 16.6 mM h-SDS in water (I_110 mM h-SDS_ or I_16.6 mM h-SDS_) (no polymer present) and from 3% Pluronic F127 in water (I_3% F127_) (no surfactant present) and compared these sums ([I_110 mM h-SDS_ + I_3% F127_] or [I_16.6 mM h-SDS_ + I_3% F127_]) in Figure 5b with the scattering profiles obtained from aqueous solutions of 110 mM h-SDS or 16.6 mM h-SDS and 3% Pluronic F127 (I_110 mM h-SDS + 3% F127_ or I_16.6 mM h-SDS + 3% F127_). For both SDS concentrations considered here, the actual scattering profile originating from the h-SDS + Pluronic F127 solution is different from that calculated from the summation of the individual component scattering (h-SDS alone, I_16.6 mM h-SDS_, and Pluronic F127 alone, I_3% F127_). The 16.6 mM h-SDS + 3% Pluronic F127 solution scattering (I_16.6 mM h-SDS + 3% F127_) (green line) exhibits a correlation peak, however the sum [I_16.6 mM h-SDS_ + I_3% F127_] (blue line) does not. In the case of 110 mM h-SDS + 3% Pluronic F127 (I_110 mM h-SDS + 3% F127_), the actual correlation peak maximum appears at higher q-values compared to the sum [I_110 mM h-SDS_ + I_3% F127_]. This comparison confirms that the surfactant–polymer association alters the polymer conformation or surfactant micelle structure from their original states in water. The correlation peak suggests a polyelectrolyte behavior due to repulsive interactions between SDS molecules bound in SDS-rich SDS/Pluronic F127 assemblies.

Qualitative information on the organization of polymer-bound SDS molecules can be obtained by analyzing the scattering contribution from the surfactant incorporated in the surfactant/polymer assemblies. To this end, we have utilized contrast matching to separate the individual scattering contributions from the amphiphilic polymer and from the surfactant present in SDS/Pluronic assemblies. d-SDS has the same scattering length density as the D_2_O solvent. Therefore, in 110 mM or 16.6 mM d-SDS + hydrogenous 3% Pluronic F127 in D_2_O, the scattering originates only from the amphiphilic polymer. The scattering contribution from the surfactant in SDS/Pluronic assemblies can be obtained by subtracting I_110 mM or 16.6 mM d-SDS + 3% F127_ from I_110 mM or 16.6 mM h-SDS + 3% F127_.

As shown in Figure 5a,b, the scattering profiles of 3% Pluronic F127 and of 110 mM or 16.6 mM d-SDS + 3% Pluronic F127 (where d-SDS does not contribute to the scattering) are completely different. The scattering intensity resulting from the subtraction of I_110 mM d-SDS + 3% F127_ from I_110 mM h-SDS + 3% F127_ is almost the same as the scattering intensity of polymer-free h-SDS micelles at 110 mM in D_2_O. The peak appears at a higher q-value, indicating that the intermicelle distance in 110 mM SDS + 3% Pluronic F127 system is lower than that observed in the case of polymer-free h-SDS micelles at 110 mM in D_2_O. The scattering intensity (black line) resulting from the subtraction of I_16.6 mM d-SDS + 3% F127_ from I_16.6 mM h-SDS + 3% F127_ is very different from the scattering intensity of polymer-free h-SDS micelles at 16.6 mM in D_2_O (cyan line). The peak is sharp, and the intensity is high compared to that from polymer-free h-SDS micelles at 16.6 mM in D_2_O. I_110 mM or 16.6 mM d-SDS + 3% F127_ shows an interaction peak at the same q-value as in I_110 mM or 16.6 mM h-SDS + 3% F127_, and the two scattering curves have similar shapes in the q range considered (0.004–0.5 Å^−1^). This suggests that both h-SDS and d-SDS form similar assemblies with Pluronic F127. Similar observations have been previously reported for the surfactant + polymer mixtures SDS + Pluronic L64 (EO_13_PO_30_EO_13_) and SDS + poly(2-(dimethylamino)ethyl methacrylate) (PDMAEMA) [49,64].

I_110 mM d-SDS + 3% F127_ is lower, and the correlation peak falls at higher q-value when compared to I_16.6 mM d-SDS + 3% F127_. In I_110 mM d-SDS + 3% F127_ and I_16.6 mM d-SDS + 3% F127_, the scattering originates only from the polymer (d-SDS does not contribute to the scattering). This suggests that the 110 mM d-SDS + 3% Pluronic F127 system comprises fewer polymer molecules in a micelle and greater number of micelles in a solution, compared to the 16.6 mM d-SDS + 3% Pluronic F127 system, supporting the picture that Pluronic-rich SDS/Pluronic assemblies break down and form surfactant-rich SDS/Pluronic assemblies upon SDS addition. The above SANS data analysis is qualitative but valuable, as it is model-independent and includes contrast matching.

#### 3.2.2. Pluronic P123 Systems

The pyrene I1/I3 ratios of SDS + 0.5% Pluronic P123 solutions are shown in Figure 2b,d. The I1/I3 ratio of 0.5% Pluronic P123 aqueous solution in the absence of surfactant is 1.30 due to pyrene localizing inside Pluronic P123 micelles. The SDS + 0.5% Pluronic P123 I1/I3 ratio started to decrease at 1 mM SDS, reached an almost constant value at 10 mM, and again started to decrease above 25 mM SDS. The I1/I3 decrease at 1 mM SDS may be due to the binding of SDS to Pluronic P123 unimers to form SDS-rich SDS/Pluronic P123 assemblies, while the I1/I3 decrease above 25 mM SDS may be due to the formation of free SDS micelles. Different from the SDS + 3% Pluronic F127 system, a decrease in the I1/I3 ratio at lower SDS concentrations (<1 mM), which would have been indicative of formation of Pluronic-rich SDS/Pluronic P123 assemblies, is not observed. This may be related to the Pluronic P123 micelle structure. If SDS binding to Pluronic P123 micelles does not change the hydrophobicity in the vicinity of localized pyrene molecules, then I1/I3 should not change. Another reason could be that SDS binds to Pluronic P123 micelles at ~1 mM and forms Pluronic-rich SDS/Pluronic P123 assemblies, which break down and form SDS-rich SDS/Pluronic P123 assemblies within a narrow concentration range, unlike the SDS + Pluronic F127 system where the binding takes place gradually, spanning a wide SDS concentration range [41]. ITC results for SDS + 0.1% Pluronic P123 at 40 °C (micellized P123) show that SDS starts binding to Pluronic P123 micelles at 1 mM, Pluronic-rich SDS/Pluronic P123 assemblies break down at 1–5 mM SDS, and polymers become saturated with SDS at 18 mM, above which free SDS micelles are present in the solution [41].

Conductivity of SDS + 0.5% Pluronic P123 aqueous solutions is plotted in Figure 1b. In the SDS concentration range where the conductivity was measured, the SDS present in the 0.5% Pluronic P123 solution is mostly associated with the polymer, resulting in a gradual change in the slope of the conductivity vs. surfactant concentration curve. As a result, the SDS concentration where the slope changes cannot be accurately determined. At higher SDS concentrations, the SDS + 0.5% Pluronic P123 solution conductivity is greater than that of SDS in plain water, similar to what was observed for the SDS + 0.5% Pluronic F127 system.

The surface tension of SDS + 0.5% Pluronic P123 aqueous solutions at 22.6 °C is presented in Figure 3b,d. The surface tension started to decrease at 0.25 mM SDS, reached a plateau at 2 mM SDS, and then started to increase above 4 mM SDS. The increase in the surface tension above 4 mM SDS may be due to the formation of SDS-rich SDS/Pluronic P123 assemblies, which have a polyelectrolyte [59] nature and desorb from the air/water interface into the bulk solution. Above 10 mM SDS, the surface tension decreased again, likely due to the saturation of Pluronic P123 by SDS and increase in the SDS monomer concentration.

The relative viscosity of SDS (at 18.5 °C) in 0.001%, 0.5%, and 2.5% Pluronic P123 aqueous solutions is shown in Figure 4b,d. 0.001% is well below the CMC of Pluronic P123; 0.5% is close to the Pluronic P123 CMC at this temperature; 2.5% Pluronic P123 is well above the CMC. The relative viscosity of SDS in 2.5% Pluronic P123 at low SDS concentrations (<25 mM) remained more or less constant but, above 25 mM SDS, increased with SDS concentration. On the basis of our pyrene fluorescence and surface tension results, we believe that the SDS concentration range below 25 mM corresponds to the concentration region where SDS-rich SDS/Pluronic P123 assemblies form. In the case of SDS + 3% Pluronic F127 system, the relative viscosity also did not change much in the concentration range where SDS-rich SDS/Pluronic F127 assemblies form. Above 25 mM SDS, the relative viscosity of the SDS + 2.5% Pluronic P123 system increased. This may be due to the formation of free SDS micelles in the aqueous solution: an increase in the number of “particles” in the solution increases the relative viscosity.

In the SDS + 0.001% Pluronic P123 system, for a ~10% increase in relative viscosity following a 140% change in ϕ_dry_, the increase in both ϕ_hydr_, and [ϕ_hydr_ - ϕ_dry_] is also ~140%. This indicates that the hydration does not change, similar to SDS + 0% or 0.01% Pluronic F127 systems. Whereas in the case of SDS + 0.5% Pluronic P123, for a ~10% increase in relative viscosity (following a 110% change in ϕ_dry_), the increase in ϕ_hydr_ is 190% and in [ϕ_hydr_ - ϕ_dry_] is 270%. This greater increase in ϕ_hydr_ (1.7 times) or [ϕ_hydr_ - ϕ_dry_] (2.5 times) with respect to ϕ_dry_ in the SDS + 0.5% Pluronic P123 system likely indicates a more extended conformation when Pluronic molecules participate in SDS-rich SDS/Pluronic assemblies. This is similar to the case of SDS + 0.5% Pluronic F127 presented above.

On the basis of our pyrene fluorescence and surface tension results and also ITC results [41], we identified in the SDS + 0.5% Pluronic P123 system four SDS concentration regions of different surfactant–polymer association modes, similar to the SDS + 3% Pluronic F127 system. In region I (below 1 mM SDS), association between SDS and Pluronic P123 micelles is not detectable. In region II (1–4 mM) SDS associates with Pluronic P123 micelles to form Pluronic-rich SDS/Pluronic P123 assemblies, which decrease in size and Pluronic P123 association number with increasing SDS concentration. In region III (4–25 mM), SDS forms SDS-rich SDS/Pluronic P123 assemblies with Pluronic P123 unimers. In region IV (above 25 mM), SDS-rich SDS/Pluronic P123 assemblies co-exist with free SDS micelles in the aqueous solution.

Despite the different amphiphilic polymer concentration (1%) and temperature (40 °C) used, the previously reported [48] SDS/Pluronic P123 assemblies are consistent with the structural transition identified in the present study with increasing SDS concentration, from Pluronic-rich to surfactant-rich SDS/Pluronic P123 assemblies. The structure of SDS-rich SDS/Pluronic P123 assemblies has not been reported in the literature. SANS can help resolve the structure of Pluronic-rich and SDS-rich SDS/Pluronic P123 assemblies. Further, a comparison of SANS results for the SDS + Pluronic P123 and SDS + Pluronic F127 systems can provide information on the effect of polymer hydrophobicity on surfactant–polymer assembly structures formed.

SANS data for SDS + Pluronic P123 solutions in D_2_O for both hydrogenous and deuterated SDS were collected (Figure 5c,d). In the absence of ionic surfactant, the scattering from 0.5% Pluronic P123 does not show any correlation peak; however, a pronounced peak appears upon addition of 16.6 mM or 110 mM h-SDS, indicating electrostatic repulsion due to ionic surfactant micelle formation [59]. The 16.6 mM SDS + 0.5% Pluronic P123 composition falls in region III (4–25 mM) where SDS forms SDS-rich SDS/Pluronic P123 assemblies. The 110 mM SDS + 0.5% Pluronic P123 composition falls in region IV where SDS-rich SDS/Pluronic P123 assemblies co-exist with free SDS micelles in solution.

Similar to the SDS + Pluronic F127 system, the scattering profile originating from the actual mixtures of 110 mM or 16.6 mM h-SDS and 0.5% Pluronic P123 in aqueous solution (I_110 mM h-SDS + 0.5% P123_ or I_16.6 mM h-SDS + 0.5% P123_) is different than that obtained from the sum of the individual component scattering ([I_110 mM h-SDS_ + I_0.5% P123_] or [I_16.6 mM h-SDS_ + I_0.5% P123_]) (Figure 5d). The correlation peak in I_16.6 mM h-SDS + 0.5% P123_ (green line) is not observed in I_16.6 mM h-SDS_ + I_0.5% P123_ (purple line). This comparison suggests altered polymer conformation or surfactant micelle structure in SDS-rich SDS/Pluronic P123 assemblies from their original states in water, in addition to a polyelectrolyte behavior of Pluronic P123.

Figure 5c,d show that the scattering profile of 110 mM or 16.6 mM d-SDS + 0.5% Pluronic P123 in D_2_O, where scattering originates only from the hydrogenous polymer, is completely different from that of the hydrogenous polymer, 0.5% Pluronic P123 in D_2_O. The scattering intensity resulting from the subtraction of I_110 mM d-SDS + 0.5% P123_ from I_110 mM h-SDS + 0.5% P123_ is almost identical to I_110 mM h-SDS + 0.5% P123_, and similar to the scattering from h-SDS micelles at 110 mM in D_2_O (when no polymer is present) at intermediate and high q-values. This is attributed to free SDS micelles co-existing in the solution with SDS-rich SDS/Pluronic P123 assemblies. Unlike the case of I_110 mM d-SDS + 3% F127_, I_110 mM d-SDS + 0.5% P123_ does not exhibit a strong interaction peak. For the lower SDS concentration considered here, the scattering intensity (black line) resulting from the subtraction of I_16.6 mM d-SDS + 0.5% P123_ from I_16.6 mM h-SDS + 0.5% P123_ is very different from the scattering intensity of polymer-free h-SDS micelles at 16.6 mM in D_2_O (cyan line) but very similar to I_16.6 mM h-SDS + 0.5% P123_ (green line). The peak is rather sharp and the intensity is high compared to polymer-free h-SDS micelles at 16.6 mM in D_2_O. I_16.6 mM d-SDS + 0.5% P123_ and I_16.6 mM h-SDS + 0.5% P123_ show an interaction peak at the same q-value and have a similar shape, suggesting that assemblies of the same structure are present in both systems.

I_110 mM d-SDS + 0.5% P123_ is lower and a strong correlation peak is not observed when compared to I_16.6 mM d-SDS + 0.5% P123_. In I_110 mM d-SDS + 0.5% P123_ and I_16.6 mM d-SDS + 0.5% P123_, the scattering originates only from the polymer. This suggests that the 110 mM d-SDS + 0.5% Pluronic P123 system comprises a smaller number of polymer molecules in a micelle, and a higher number of micelles in the aqueous solution, compared to the case of 16.6 mM d-SDS + 0.5% Pluronic P123, supporting the picture that Pluronic-rich SDS/Pluronic assemblies break down and form SDS-rich SDS/Pluronic assemblies upon surfactant addition.

### 3.3. Comparison of Systems Containing Pluronic F127 and Pluronic P123 below the CMC

Below the CMC of the amphiphilic polymer, the mode of association of SDS and PEO-PPO-PEO is the same for both Pluronic F127 and Pluronic P123. The SDS concentration range where SDS-rich SDS/Pluronic assemblies form is almost the same for both Pluronic F127 (0.01%) and Pluronic P123 (0.001%). The critical association concentration (CAC) of SDS in 0.001% unassociated Pluronic P123 solution (~1 mM) is very close to the CAC of SDS in 0.01% unassociated Pluronic F127 solution (~0.5 mM), and the CAC values are much smaller than the CAC of SDS in the presence of a PEO homopolymer. In SDS + Pluronic F127 solutions, a Pluronic concentration variation in the range 0.01–0.5% did not affect the CAC value [36].

In order to understand the individual contributions of the PPO and PEO blocks on SDS/PEO-PPO-PEO block copolymer interactions, we consider aqueous solutions of SDS with PEO or PPO homopolymers. In aqueous PEO homopolymer solutions, the CAC of SDS decreased from 6 mM for PEO 600 molar mass and reached a constant value of 4.2 mM for PEO above 4600 molar mass [65,66]. The CAC of SDS in aqueous PEO solutions was found independent of polymer concentration (in the range 0.01–0.1 wt%) [66]. Due to the limited aqueous solubility of PPO, aqueous SDS + PPO solutions have been studied for only short-chain PPO with an average molar mass 1000 (PO_14_). For SDS in aqueous PPO 1000 solutions, CAC decreased from 2.7 mM to 1 mM with increasing PPO concentration from 0.05% to 0.5% [67,68]. These values show that, for the same PEO and PPO average molar mass, the CAC of SDS in aqueous PPO solutions is lower than that in PEO solutions. Pluronic F127 and P123 have the same number of PO segments, but Pluronic F127 has 80 EO segments more in each PEO block compared to Pluronic P123. The above observations indicate that the PPO blocks in PEO-PPO-PEO block copolymers mainly influence SDS binding to unassociated polymers. A similar observation has been made previously [41]. This could suggest a stronger interaction of SDS with the hydrophobic PPO compared to PEO. As attested to by the SANS and surface tension results presented here, the SDS-rich SDS/Pluronic assemblies exhibit polyelectrolyte nature for both Pluronic F127 and P123.

### 3.4. Comparison of Systems Containing Pluronic F127 and Pluronic P123 above the CMC

Some notable differences are observed in the mode of interaction at SDS–Pluronic P123 and SDS–Pluronic F127 systems above the CMC of the amphiphilic polymers. SDS binding to Pluronic F127 micelles commences at a much lower surfactant concentration (< 0.01 mM) compared to that of Pluronic P123 (~1 mM), indicating that SDS has a stronger binding affinity to Pluronic F127 micelles. ITC has also shown that SDS binds stronger to Pluronic F127 micelles than to Pluronic P123 micelles, however the reason for this stronger binding was reported to be unknown [41]. The enthalpy change for the formation of SDS-rich SDS/Pluronic assemblies (region III) from block polymer micelles and surfactant micelles reported per mole of polymer, was more exothermic for Pluronic F127 (–134 kJ/mol) compared to Pluronic P123 (–100 kJ/mol) [41]. To calculate the enthalpy change per polymer segment, we considered the two extreme cases: (i) the total enthalpy change is due to EO segments (PO segment contribution is negligible), and (ii) both EO and PO segments contribute (in an equal manner) to the total enthalpy change. In the first case, the enthalpy change is –0.67 kJ/(mol EO segment) for Pluronic F127 and –2.63 kJ/(mol EO segment) for Pluronic P123. In the second case, the enthalpy change is –0.51 kJ/(mol EO or PO segment) for Pluronic F127 and is –0.93 kJ/(mol EO or PO segment) for Pluronic P123. The enthalpy change per polymer mass is –10.6 kJ/kg for Pluronic F127 and –17.4 kJ/kg for Pluronic P123. On the basis of the above numbers, Pluronic P123 clearly “wins” on a per-segment and per-mass basis, however Pluronic F127 wins per whole molecule.

The stronger binding of SDS to Pluronic F127 micelles compared to Pluronic P123 micelles might be due to either difference in the Pluronic micelle structure or difference in the length of PEO chains. The CAC of SDS in 0.1% PEO 8000 (EO_181_) solution is 4.2 mM compared to 5.9 mM in 0.1% PEO 900 (EO_20_) solution [66]. Since Pluronic micelles comprise PPO chains inside the core and PEO chains in the outer shell, SDS binds to Pluronic F127 micelles with EO_100_ chains in the shell at a lower SDS concentration compared to Pluronic P123 micelles with EO_19_ chains in the shell. A previous study reported that SDS binds to Pluronic L121 (EO_5_PO_69_EO_5_) micelles at 1.5 mM [41]. Pluronics F127, P123 and L121 all have the same PPO block length but different PEO block lengths. The concentration where SDS binds to Pluronic block copolymer micelles increased with decreasing number of EO segments [41]. However, for Pluronic L64 (EO_13_PO_30_EO_13_), even though it has a smaller number of EO segments, it was reported that SDS formed Pluronic-rich SDS/Pluronic assemblies at very low SDS concentrations (<0.01 mM) [49]. Hence, it is difficult to rationalize why SDS binds to Pluronic F127 (EO_100_PO_65_EO_100_) micelles at very low concentrations (< 0.01 mM) compared to Pluronic P123 (EO_19_PO_69_EO_19_) micelles (1 mM) based on PPO/PEO composition. The core-shell sphere structure is the same for both Pluronic F127 and Pluronic P123 micelles, however the scattering profile of Pluronic P123 shows a side maximum that was not observed in the Pluronic F127 scattering profile (Figure 5). This side maximum is expected to result from the form factor of dense spherical objects with a sharp interface, in this case, the core-shell interface between PPO and PEO [69]. This could be a reason for stronger binding of SDS to Pluronic F127 micelles compared to Pluronic P123 micelles.

Upon SDS addition to aqueous solution of Pluronic micelles, an exotherm was observed in ITC, which was ascribed to the break down of Pluronic micelles [41,48]. The exotherm was more negative for higher Pluronic concentrations (–12 kJ/mol for SDS + 0.1% P123 at 40 °C and –27 kJ/mol for SDS + 1% Pluronic P123 at 40 °C) [41,48]. However, an endothermic peak is observed in the case of the PEO homopolymer when SDS-rich SDS/Pluronic assemblies form upon SDS addition [65]. This endotherm increased with increasing PEO molar mass (at 0.1 wt%) and reached an almost constant value (~4.5 kJ/mol) for PEO molar mass greater than 4600 [65]. At the end of the exothermic peak in ITC curves, where Pluronic micelles have been completely disintegrated, the molar ratio of surfactant to polymer (N_s_/N_p_) indicated that ~9 SDS molecules per polymer chain were required to completely break down the Pluronic F127 micelles, whereas 22 SDS molecules were required for Pluronic P123 micelles [41]. Pluronic P123 micelles have a greater association number compared to Pluronic F127 micelles, hence a higher number of SDS molecules would be required to completely break down a Pluronic P123 micelle.

The SDS-rich SDS/Pluronic F127 assemblies form over a wider SDS concentration range (region III spans 2.5–100 mM SDS) when compared to SDS-rich SDS/Pluronic P123 assemblies (region III: 4–25 mM SDS). Free SDS micelles form at higher SDS concentrations in 3% Pluronic F127 solution when compared to 0.5% Pluronic P123 solution. This may be due to the greater number (nearly thrice) of Pluronic F127 molecules in the 3% (2.38 mM) Pluronic F127 solution compared to the number of Pluronic P123 molecules in the 0.5% (0.87 mM) Pluronic P123 aqueous solution; the amount of surfactant required to saturate the polymers in SDS-rich SDS/Pluronic assemblies will be higher in the case of 3% Pluronic F127.

Comparing the mode of interaction of SDS (CMC = 8.7 mM in pure water in the absence of block copolymers) with PEO-PPO-PEO block copolymers Pluronic F127 and P123 above and below the Pluronic CMC (in the absence of surfactant), we can observe that SDS starts binding to Pluronic F127 micelles at a much lower concentration (~0.01 mM) when compared to the unassociated Pluronic F127 (~0.5 mM). Whereas in the case of Pluronic P123, the concentration at which SDS binds to unassociated Pluronic P123 is close to that of micellized Pluronic P123 (~1 mM). The CAC of SDS in aqueous PEO homopolymer solutions [65,66] is much higher than the CAC of SDS in aqueous Pluronic solutions below the CMC. This shows that the amphiphilic polymer hydrophobicity greatly influences the interactions between ionic surfactants and amphiphilic polymers. More specifically, ionic surfactants are more difficult to bind to homopolymers, compared to amphiphilic block copolymers. The PEO chain length may have a minor effect on the SDS CAC below the CMC of PEO-PPO-PEO block copolymers. However, the very small CAC values (in the range 0.5–1 mM) and the differences in the CAC values obtained by different experiment techniques make it difficult to rationalize the PEO chain length effect on CAC below the Pluronic CMC.

## 4. Conclusions

Amphiphilic polymers can associate in aqueous solutions to form micelles in a manner analogous to that of short-chain surfactants. Such micelles are expected to alter the mode of interaction between long-chain and short-chain amphiphiles. In this study, we investigated the interactions between the common ionic surfactant SDS and nonionic poly(ethylene oxide)–poly(propylene oxide)–poly(ethylene oxide) (PEO-PPO-PEO) amphiphilic polymers (Pluronics or Poloxamers), at block copolymer concentrations below and above their CMC in plain water. The macromolecular amphiphiles Pluronic F127 (low PPO/PEO ratio) and Pluronic P123 (high PPO/PEO ratio) have the same number of PO segments but different numbers of EO segments; this renders Pluronic P123 more hydrophobic compared to Pluronic F127. Fluorescence and surface tension techniques used in this study complement each other in assessing the surfactant–polymer interactions and in determining SDS concentration regions in which different modes of surfactant–polymer association take place. SANS with contrast variation provides evidence on the nature of SDS/Pluronic assemblies.

**Figure 6:**
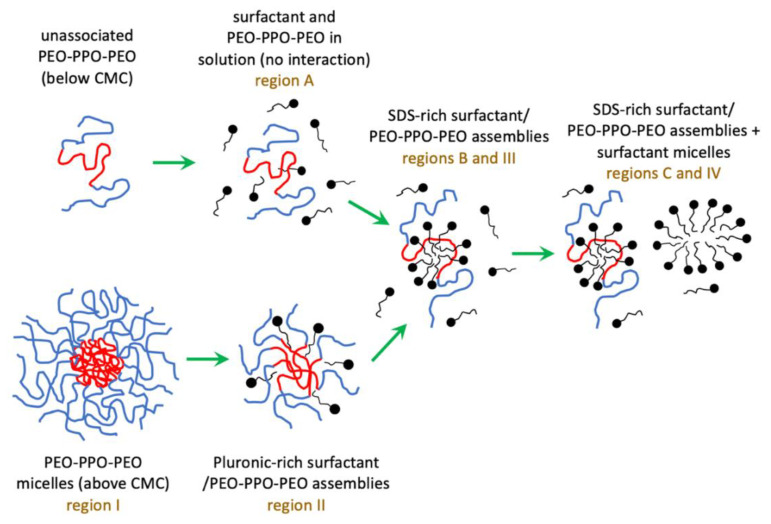
Different modes of PEO-PPO-PEO block copolymer and ionic surfactant interactions in aqueous solution when an increasing amount of ionic surfactant is added to polymer solutions of fixed concentration (below or above the block copolymer CMC in plain water).

Below the CMC of the macromolecular amphiphiles, their mode of interaction with SDS is as follows (Figure 6): PEO-PPO-PEO molecules compete with SDS molecules to adsorb at the air/liquid interface (region A), SDS-rich SDS/Pluronic assemblies form above the CAC (region B), followed by formation of polymer-free SDS micelles (region C). Below their CMC, Pluronic F127 and Pluronic P123 interact with SDS in a similar mechanism by forming SDS-rich SDS/Pluronic assemblies that have a polyelectrolyte nature, as attested by the presence of a correlation peak in SANS and an increase in the surface tension. The SDS concentration range where SDS-rich SDS/Pluronic assemblies form is almost the same for both polymers, and the CAC or C_m_ values of SDS are similar in both Pluronic F127 and Pluronic P123 solutions.

Above the CMC of the macromolecular amphiphiles, with the binding of SDS, the Pluronic micelles (region I) decrease in size and association number and form Pluronic-rich SDS/Pluronic assemblies (region II). Upon further increase in SDS concentration, these Pluronic-rich assemblies transition into SDS-rich SDS/Pluronic assemblies (region III) and, when the Pluronic molecules become saturated with SDS, free SDS micelles form in the aqueous solution (region IV) (Figure 6). A comparison between d-SDS + Pluronic F127 or P123 SANS data at 110 mM and 16.6 mM d-SDS concentrations supports the picture that Pluronic-rich SDS/Pluronic assemblies break down and form surfactant-rich SDS/Pluronic assemblies upon the addition of surfactant. Above the CMC of the macromolecular amphiphiles, the difference in the hydrophobicity of polymers resulted in some differences in the mode of association between SDS and Pluronic. SDS molecules bind to Pluronic F127 micelles at a much lower surfactant concentration (~0.01 mM) when compared to Pluronic P123 micelles (~1 mM). This could be due to a stronger interaction of SDS with longer PEO chains in Pluronic F127 (EO_100_PO_65_EO_100_) compared to Pluronic P123 (EO_19_PO_69_EO_19_), and/or differences in the micelle core/corona composition between Pluronic F127 and Pluronic P123 micelles. For the Pluronic concentrations considered here, the SDS-rich SDS/Pluronic assemblies form over a wider SDS concentration range (region III), and free SDS micelles form at higher SDS concentrations in an SDS + Pluronic F127 system when compared to an SDS + Pluronic P123 system.

The results presented here demonstrate that the polymer hydrophobicity and concentration in aqueous solution can have a great influence on the polymer interactions and organization with ionic surfactants in surfactant+polymer mixed systems. A combination of experimental techniques provides direct evidence on the nature of assemblies formed between SDS and PEO-PPO-PEO amphiphilic polymers with low and high PEO/PPO ratio. Detailed analysis of the SANS intensity data will be presented elsewhere. SANS data on SDS/Pluronic P123 assemblies are not available in the literature, and contrast matching has not been previously reported for SDS-rich SDS/Pluronic F127 assemblies. This study provides fundamental insights on amphiphilic polymer and ionic surfactant organization in aqueous solutions that benefit the diverse applications of complex fluids.
